# Response to selection for parasitism of a suboptimal, low‐preference host in an aphid parasitoid

**DOI:** 10.1111/eva.13254

**Published:** 2021-06-21

**Authors:** Keith R. Hopper, Kameron T. Wittmeyer, Kristen L. Kuhn, Kathryn Lanier

**Affiliations:** ^1^ Beneficial Insect Introductions Research Unit USDA‐ARS Newark DE USA

**Keywords:** genomics, host adaptation, Hymenoptera, parasitoid, quantitative genetics, response to selection

## Abstract

Risks of postintroduction evolution in insects introduced to control invasive pests have been discussed for some time, but little is known about responses to selection or genetic architectures of host adaptation and thus about the likelihood or rapidity of evolutionary shifts. We report here results on the response to selection and genetic architecture of parasitism of a suboptimal, low‐preference host species by an aphid parasitoid, *Aphelinus rhamni*, a candidate for introduction against the soy bean aphid, *Aphis glycines*. We selected *A*. *rhamni* for increased parasitism of *Rhopalsiphum padi* by rearing the parasitoid on this aphid for three generations. We measured parasitism of *R*. *padi* at generations 2 and 3, and at generation 3, we crossed and backcrossed parasitoids from the populations reared on *R*. *padi* with those from populations reared on *Aphis glycines* and compared parasitism of both *R*. *padi* and *Aphis glycines* among *F*_1_ and backcross females. *Aphelinus rhamni* responded rapidly to selection for parasitism of *R*. *padi*. Selection for *R*. *padi* parasitism reduced parasitism of *Aphis glycines*, the original host of *A*. *rhamni*. However, parasitism of *R*. *padi* did not increase from generation 2 to generation 3 of selection, suggesting reduced variance available for selection, which was indeed found. We tested the associations between 184 single nucleotide polymorphisms (SNP) and increased parasitism of *R*. *padi* and found 28 SNP loci, some of which were associated with increased and others with decreased parasitism of *R*. *padi*. We assembled and annotated the *A*. *rhamni* genome, mapped all SNP loci to contigs and tested whether genes on contigs with SNP loci associated with parasitism were enriched for candidate genes or gene functions. We identified 80 genes on these contigs that mapped to 1.2 Mb of the 483 Mb genome of *A*. *rhamni* but found little enrichment of candidate genes or gene functions.

## 1 INTRODUCTION

Invasions by exotic species that become pests are an increasing problem for agriculture (Bradshaw et al., 2016). Biological control by introduction of natural enemies has proved effective at reducing the abundance and impact of such pests (Cock et al., 2016), and in principle, provides a safe, cost‐effective, and sustainable alternative to widespread application of insecticides (Naranjo et al., 2015). In spite of notable successes, two arguments have been made against the introduction of natural enemies for biological control. The first is that introduced natural enemies may attack native nontarget species and reduce their abundance (Simberloff & Stiling, 1996). Modern methods of host‐specificity screening prior to introduction are designed to ensure that organisms introduced for the control of insect (Hoddle, 2005) and weed (McEvoy, 1996) pests do not attack native nontarget species, and thus, the immediate threat of nontarget impacts is greatly reduced. However, a related concern has not been adequately addressed: the possibility that postintroduction evolution in host specificity may result in introduced organisms shifting to attack native nontarget species (Simberloff & Stiling, 1996). If host use can evolve rapidly, then traditional screening will not guarantee the safety of biological control introductions. The second argument against such introductions is that native natural enemies may switch to attack invading pests. Populations of invasive species freed from their native natural enemies can quickly achieve high densities, which may cause behavioural or evolutionary shifts by native natural enemies to attack the invaders. Because of such switches, control of the invading pest may eventually be achieved without the introduction of non‐native biological control agents (Carroll et al., 2005; Cox, 2004; Kruitwagen et al., 2018).

Although these issues have been discussed for some time, responses to selection and genetic architectures of host adaptation have been studied in few systems (however, see Auer et al., 2020; McBride et al., 2014; Oppenheim et al., 2012, 2018), and thus, little is known about the likelihood or rapidity of evolutionary shifts in host adaptation after introductions for biological control. The existing evidence concerning postintroduction evolution in host adaptation is weak and controversial (Marohasy, 1996; Secord & Kareiva, 1996; van Klinken & Edwards, 2002). For parasitoids, much is known about their foraging and host selection behaviour (Godfray, 1994; Hoddle, 2005; Strand & Obrycki, 1996), but the evolutionary stability of parasitoid host adaptation is unclear and has received scant attention (Hopper et al., 2005; Hufbauer & Roderick, 2005). The working hypothesis among biological control researchers is that evolutionary changes in host adaptation are genetically complex and therefore unlikely (Hopper et al., 1993). This hypothesis is largely untested: little is known about the genetic architecture of host adaptation for most insects, let alone about the effects of genetic architecture on evolutionary shifts (Hopper et al., 1993, 2005; Hufbauer & Roderick, 2005; Oppenheim et al., 2012; Oppenheim & Hopper, 2010). The host ranges of parasitoids may be affected by genes underlying a variety of processes, including the ability of female parasitoids to find insects and to recognize and oviposit in those found, as well as subsequent survival of parasitoid progeny in hosts (Vinson, 1976). Oviposition may be limited by parasitoid decisions (host acceptance) or insect defences, including behavioural and structural defences (Gross, 1993), as well as ecological defences (like ant tending and plant chemistry). Survival after oviposition (host suitability) depends on adequate nutrition and may be affected by synchrony with host development, physiological suppression, host–plant chemistry, and immune responses (for review, see papers in special issue of Journal of Insect Physiology 44 (9): 701–866). Survival may also be affected by bacterial endosymbionts in hosts (Asplen et al., 2014; Hopper et al., 2018; Oliver et al., 2003, 2005). From an applied perspective, predicting the likelihood of evolution in host adaptation depends on knowledge of how many genes are involved and how they interact (Oppenheim et al., 2012, 2018): phenotypes that depend on a few genes that interact additively are far more likely to respond rapidly to selection than ones that depend on many genes that interact epistatically. The importance of genetic architecture for response to selection is supported by the results on both the evolution of insecticide resistance (Hardstone & Scott, 2010) and the breakdown of plant resistance to pest insects and diseases (Harris et al., 2003; Neuhauser et al., 2003).

We report here the results on the response to selection for parasitizing a suboptimal, low‐preference host species by an aphid parasitoid, *Aphelinus rhamni* Hopper and Woolley (Hymenoptera: Aphelinidae), and the genetic architecture of this response. *Aphelinus rhamni* is being introduced to control *Aphis glycines*, which has become a major pest of soya bean in North America (Hopper, Lanier, Rhoades, Hoelmer, et al., 2017). In previous research on the laboratory colony studied here, *A*. *rhamni* females parasitized *Rhopalosiphum padi* (L.) (Hemiptera: Aphididae) three times less frequently than *Aphis glycines* Matsumura (Hemiptera: Aphididae), and this difference arose from differences in both female behaviour and progeny survival (Hopper, Lanier, Rhoades, Hoelmer, et al., 2017). In that research, 25‐minute observations of *A*. *rhamni* females exposed to *R*. *padi* versus *Aphis glycines* showed that the parasitoids approached fewer *R*. *padi* (3.5 vs. 6.3 aphids), probed fewer *R*. *padi* with their ovipositors (2.7 vs. 5.1 aphids) and laid eggs in fewer *R*. *padi* (0.4 vs. 2.6 aphids). While the difference in oviposition was fivefold between *R*. *padi* and *Aphis glycines*, the difference in numbers of adult progeny was 11‐fold, suggesting mortality of about 50 per cent for eggs laid in *R*. *padi*. However, some *A*. *rhamni* females parasitized as many as seven *R*. *padi* per day and produced as many as five adult progeny per day, suggesting that there might be genetic variation in use of *R*. *padi*. To explore this possibility, we selected *A*. *rhamni* for increased preference and performance on *R*. *padi* by rearing the parasitoid species on this aphid for three generations. We measured parasitism of *R*. *padi* at generations 2 and 3, and at generation 3, we crossed and backcrossed parasitoids from the populations reared on *R*. *padi* with those from the populations reared on *Aphis glycines* and compared parasitism of both *R*. *padi* and *Aphis glycines* among *F*_1_ and backcross females. We determined the relationship between genetic markers and parasitism. Finally, we assembled and annotated the *A*. *rhamni* genome and tested whether genes near genetic markers were enriched for candidate genes or classes of gene functions. Our results show a genetically based response to selection on *A*. *rhamni* for parasitism of *R*. *padi* with a complex architecture. Our results imply that parasitoids introduced for biological control may rapidly evolve to attack nontarget species but that this evolution may be limited. As mentioned above, *Aphelinus rhamni* is being introduced to control *Aphis glycines*, a major invasive pest of soya bean, and our results suggest that it may shift to attack other aphid species somewhat.

## 2 MATERIALS AND METHODS

### 2.1 Study system

*Aphelinus* species are important in biological control of pest aphids (Hopper, Lanier, Rhoades, Coutinot, et al., 2017; Hopper, Lanier, Rhoades, Hoelmer, et al., 2017; van den Bosch et al., 1959). Like all *Aphelinus* species, *A*. *rhamni* is a koinobiont (host continues to develop after being parasitized) endoparasitoid of aphids. *Aphelinus* species are small (about 1 mm long) and are weak fliers (Fauvergue & Hopper, 2009), searching for hosts and mates primarily while walking (Fauvergue et al., 1995). *Aphelinus* females prefer 2nd‐ to 4th‐instar aphids for oviposition, but will oviposit in all stages (Rohne, 2002). At 20℃, wasps develop from oviposited egg to adult emergence in about three weeks. During their third instar, *Aphelinus* larvae kill their hosts, but leave the host exoskeleton intact, causing it to harden and turn black in a process called mummification (Christiansen‐Weniger, 1994), and adults emerge about one week after pupation. *Aphelinus rhamni* females 1–2 days old carry a mean of 15 mature eggs (Hopper, Lanier, Rhoades, Hoelmer, et al., 2017), but females can produce more eggs daily and so could parasitize as many as 200 aphids during a two‐week lifetime (unpublished data).

### 2.2 Aphids and host plants

Aphids for parasitoid rearing and experiments were from laboratory cultures at USDA‐ARS Beneficial Insect Introduction Research Unit, Newark, Delaware (BIIRU), started with aphids from field collections near Newark. The culture of *R*. *padi* was started in 1997 and is reared on barley, *Hordeum vulgare* L. variety Lacey, and the culture of *Aphis glycines* was started in 2004 and is reared on soy bean, *Glycine max* (L.) variety Pioneer 91Y70. The aphids were screened for the secondary, bacterial endosymbionts *Arsenophonus*, *Hamiltonella* and *Regiella* with PCR and primers specific to these bacteria, but the only endosymbiont found was *Arsenophonus* in *A*. *glycines* (unpublished data), which in other research has been found not to affect parasitism (Wulff et al., 2013). Aphids were reared on their host plants in plant growth rooms at ~20℃, 50%–70% relative humidity and 16:8‐h (L:D) photoperiod. Vouchers for these populations are stored at −20℃ in 100% molecular‐grade ethanol at BIIRU.

### 2.3 Collections and culture of *Aphelinus rhamni*

*Aphelinus rhamni* was collected as parasitized *Aphis glycines* on *Rhamnus* spp. in China during September 2005. The parasitoids were hand‐carried as mummified aphids to the USDA‐ARS containment facility at BIIRU under APHIS‐PPQ permit P526P‐05‐214, and the culture has been continued under permits P526P‐08‐02142, P526P‐11‐02202, P526P‐12‐02833, P526P‐15‐04273 and P526P‐18‐0465. The material was initially screened for hyperparasitoids and pathogens, and a culture was established with seven female and seven male adults for a total of 21 haplotypes, although a limited sample of this number of haplotypes is likely to have captured much of the genetic variation in fitness components (Roush & Hopper, 1995). To maintain genetic variation (Hopper et al., 1993; Roush & Hopper, 1995), the culture was split into four subcultures after one generation, and each subculture has been renewed each generation with an adult population size >200. Sex ratios of emerging adults are about 1:1 males to females. Parasitoids are reared on *A*. *glycines* on soy bean or *R*. *padi* on barley infested with several thousand aphids in cages (10 cm diameter by 22 cm tall) enclosing the foliage of potted plants in plant growth chambers (AR66‐2L; Percival Scientific) at 20℃, 50%–70% relative humidity and 16:8‐h (L:D) photoperiod. When the response to selection experiment was started, the parasitoids had been in culture 5.5 years or 90 generations. Vouchers for the selection and control populations of *A*. *rhamni* are maintained at −20℃ in 100% molecular‐grade ethanol at BIIRU.

### 2.4 Selection regime

To select *Aphelinus rhamni* for increased preference and performance on *R*. *padi*, we put ~200 adult parasitoids into each of three cages (10 cm diameter by 22 cm tall) enclosing the foliage of potted barley infested with several thousand *R*. *padi*. For three generations, we transferred ~200 adult parasitoids from each cage to a new cage with aphid‐infested barley. Thus, we produced three populations of parasitoids that were exposed to hard selection for three generations: genotypes of females that did not lay eggs in *R*. *padi* and genotypes of progeny that did not survive in *R*. *padi* would not be represented in the next generation.

### 2.5 Crosses

After three generations of selection, we crossed females from the control populations (i.e., reared on *Aphis glycines*) with males from the selection populations (i.e., reared on *R*. *padi*) to produce *F*_1_ females, which we reared on *Aphis glycines* to avoid conditioning or selection for preference/performance on *R*. *padi*. We measured parasitism of *R*. *padi* and *Aphis glycines* by these *F*_1_ females and backcrossed 29 *F*_1_ females with males from the control populations, which would produce backcross females with genotypes that were homozygous control or heterozygous control/selection. We measured parasitism of *R*. *padi* and *Aphis glycines* by exposing them to 387 backcross females and then genotyped 372 backcross females that were recovered live (Table 1).

**TABLE 1:** Sample sizes for estimates of parasitism by control, selection, *F*_1_ and backcross females of *Aphelinus rhamni*

Aphid species	Parasitoid population	Tested	Recovered	Per cent recovered
*Rhopalosiphum padi*	Generation 2: Control	20	17	85
	Generation 2: Selection	100	95	95
	Generation 3: Control	20	19	95
	Generation 3: Selection	100	91	91
*Aphis glycines*	*F*_1_ (C ♀ × S3 ♂)	57	54	95
*Rhopalosiphum padi*	*F*_1_ (C ♀ × S3 ♂)	60	57	95
	Generation 4: Control	20	19	95
	Generation 4: Selection	40	38	95
*Aphis glycines*	Backcross (*F*_1_ ♀ × C ♂)	107	89	83
*Rhopalosiphum padi*	Backcross (*F*_1_ ♀ × C ♂)	387	372	96

### 2.6 Measurement of parasitism

We measured parasitism of *R*. *padi* by 100 females of *A*. *rhamni* from selection populations and by 20 females from control populations for generations 2 and 3 of selection and parasitism of *R*. *padi* and *Aphis glycines* by 60 and 57 *F*_1_ females, respectively, and 387 and 107 backcross females, respectively. Lastly, we measured parasitism of *Aphis glycines* by 28 females from control populations. We measured parasitism of *Aphis glycines* to determine whether improved performance on *R*. *padi* correlated with reduced performance on *Aphis glycines*.

To measure parasitism, we exposed individual female parasitoids to *R*. *padi* or *Aphis glycines*. We used females that were 1–5 days old and had been with males and aphids since emergence and thus had the opportunity to mate, host feed and oviposit. To ensure that females had a full egg load, we isolated females from aphids for 24 hours before using them in experiments. We put each female in a cage (10 cm diameter by 22 cm tall) enclosing the foliage of potted plants with 100 aphids of mixed instars. Female parasitoids were removed either after 24 hours for *R*. *padi* or after seven days for *Aphis glycines*. Ten days later, we collected any mummified aphids and held them for adult parasitoid emergence. After the adults emerged, we recorded the number of mummified aphids and the number of adult–parasitoid progeny.

Because *A*. *rhamni* females carry about 15 eggs, which they can replace in one day, the abundance of aphids and period of exposure allowed parasitoids to use their full egg complement. Furthermore, the density of aphids, amount of plant material and cage size meant that parasitoids were unlikely to be limited by search rate. Therefore, we measured a combination of acceptance of hosts for oviposition and suitability of hosts for parasitoid development.

### 2.7 Analysis of parasitism and adult emergences

Replicates in which females were not recovered or died before the end of the exposure period were not included in analyses because of the risk that they were exposed to aphids for longer or shorter periods than the recovered females. This left 83–96 per cent of females tested (Table 1).

We used generalized linear models (GLMs) to test the effects of selection on the number of parasitized (mummified) aphids of each species. Although we collected data on adult emergence rates (proportion of parasitized aphids from which adult wasps emerged), there were too few *R*. *padi* parasitized by control population females to compare rates between treatments. The experimental unit for these analyses was a female parasitoid exposed to a single aphid species. These variables could have non‐normal distributions with variances proportional to means, so we used the appropriate error distribution (e.g., normal, negative binomial) for each analysis. We chose the distribution that gave highest model probability calculated from the residual deviance divided by residual degrees of freedom compared with a chi‐square distribution (Littell et al., 1996). The negative binomial distribution gave the best fit for the numbers of parasitized aphids. For these analyses, we used the GLM.NB function in the MASS R package (version 7.3‐48; Venables & Ripley, 2002) and the glm function in the STATS package in R. We calculated least‐squares means and 95% asymptotic confidence intervals using the LSMEANS function in the EMMEANS R package (version 2.27‐61; Lenth, 2016). The confidence intervals were sometimes asymmetrical so we report means and asymptotic 95% confidence levels in the following format: mean [lower confidence level − upper confidence level].

Because selection may erode genetic variance, we compared variances for the females from selection generation 2 and 3 using the *F*‐ratio in the var.test function in the R stats package (R_Core_Team, 2020). We also compared variances among females from selection generation 3, control females and backcross females because heterosis may increase variance among backcross individuals, compared with variances among their progenitors.

### 2.8 SNP discovery and genotyping

To generate single nucleotide (SNP) markers for analysis of their association with parasitism, we made and sequenced reduced‐representation libraries (RRL) of each backcross female. Such libraries can provide large numbers of sequence polymorphisms across many individuals at low cost (Baxter et al., 2011). To make these libraries, we modified a protocol from Baird et al. (2008). Genomic DNA was extracted from individual wasps using Qiagen DNeasy Blood and Tissue Kits and then whole‐genome amplified (WGA) with REPLI‐g Kits (Qiagen), following kit protocols. An aliquot (1 μg) of the resulting DNA was digested with restriction endonucleases using one rare cutter (e.g., *NgoMIV* with a 6 bp recognition site) and one frequent cutter (e.g., *CviQI* with a 4 bp recognition site), which together determine the number and locations of fragments across the genome and the lengths of these fragments. Custom adaptors, with barcodes for each sample that also served to register clusters on the Illumina HiSeq platform during sequencing, were ligated onto the fragments using T4 ligase. The ligation products were pooled and then purified using the Qiagen QIAquick PCR Purification Kit (Qiagen). The pooled ligate was size‐selected (300–350 bp) using the BluePippin System (Sage Science). The size‐selected ligate was PCR‐amplified to both increase copy number at each locus and add more adaptor sequence. The adaptors were designed so only fragments with the rare‐common combination of cut sites would amplify. After PCR, the product was purified using Agencourt AMPure XP Beads (Beckman Coulter), quantitated with qPCR and sequenced on an Illumina HiSeq 2500 (Illumina) at the Delaware Biotechnology Institute, Newark, Delaware.

### 2.9 Analysis of associations of phenotypes with SNP loci

Reduced‐representation libraries reads were cleaned and trimmed for quality using Trimmamotic (Bolger et al., 2014). The reads were then aligned to a draft genome of *A*. *rhamni* using bwa (Li & Durbin, 2009). SNP calling was done with MPILEUP in SAMtools (Li, 2011). SNP loci were filtered to retain only those with two alleles and recoded as either AA or AB. Loci were filtered to remove those called in <150 backcross females, duplicates (i.e., those with exactly the same pattern in all backcross females) and those with segregation that deviated significantly from 50 per cent homozygous control and 50 per cent heterozygous control/selection. Also, individuals with more than 50 per cent missing data were dropped. Filtering and genetic mapping of SNP loci were done with R/QTL (Broman et al., 2003). Analysis of the association between each SNP locus and numbers of parasitized *R*. *padi* was done with generalized linear models with a negative binomial error distribution using GLM.NB function from the MASS R package (version 7.3‐48; Venables & Ripley, 2002), and correction for multiple testing was done with the Benjamini and Hochberg method (Benjamini & Hochberg, 1995).

### 2.10 Genome assembly and annotation

For de novo assembly of the genome of *A*. *rhamni*, we used an Illumina paired‐end library (~300 bp inserts with 2 × 150 nt sequencing in one Illumina channel) prepared and sequenced with standard with kits and protocols from Illumina (Illumina). We assembled the genome with MaSuRCA (Zimin et al., 2013) and evaluated the genome assembly with the quantiles of contig sizes, by comparing assembly size to that estimated from flow cytometry, and by comparing gene content with the core insect gene set in BUSCO (Simão et al., 2015).

Using AUGUSTUS with the *Nasonia* gene model (Stanke & Morgenstern, 2005), we identified protein‐coding regions in the *A*. *rhamni* genome assembly. To confirm that these genes were transcribed, we mapped RNAseq data to the putative genes using Magic‐BLAST (Boratyn et al., 2019). To discover the function of these genes, we compared their amino acid sequences to proteins in the RefSeq database (accessed on 4/21/2018; ncbi.nlm.nih.gov) using BLASTP (with the BLOSUM62 scoring matrix, *E*‐value = 0.001 and the default values for other parameters) (Altschul et al., 1990) and searched for functional information using BLAST2GO (Conesa et al., 2005) and domain analyses with InterProScan (version 5; Jones et al., 2014).

### 2.11 Functions of genes near loci associated with *R*. *padi* parasitism

To explore the relationship between candidate genes and gene functions and genetic markers associated with parasitism of *R*. *padi*, we mapped all 184 SNP loci, whether associated with parasitism or not, to the contigs in our *A*. *rhamni* assembly. We then separated the contigs into sets that had loci associated with parasitism at FDR ≤0.05, and those that had loci not associated with parasitism at FDR >0.05 or FDR >0.20, and identified the genes on these sets of contigs. This filtering was done with custom scripts in R (R_Core_Team, 2020). We identified the genes in these sets of contigs, searched for candidate genes (i.e., those coding for venom proteins, cytochrome p450 proteins and chemosensory proteins) and determined whether the sets differed in the numbers of candidate genes. We also tested for enrichment of biological processes or molecular functions between the gene sets using Fisher's exact test (false discovery rate = 0.05) in BLAST2GO.

## 3 RESULTS

### 3.1 Response to selection

**FIGURE 1:**
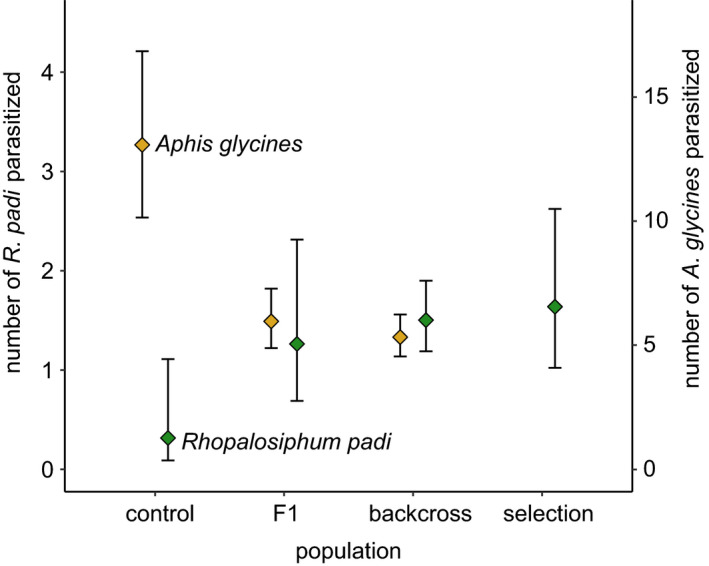
Parasitism of *Rhopalosiphum padi* by *Aphelinus rhamni* females from populations reared three generations on *R*. *padi*, control populations reared continuously on *Aphis glycines*, the *F*_1_ females from a selection population male mated with control population females and backcross females from F1 females mated with control population males. Points are the mean numbers of parasitized aphids, and vertical lines are asymptotic confidence intervals for those means

In generations 2 and 3, *A*. *rhamni* females from the selected populations parasitized 6 and 8 times more *R*. *padi* than those from the control populations, but parasitism did not increase between generations 2 and 3 (Figure 1; Table 2). Surprisingly, 40 to 50 per cent of females from the selected populations failed to produce any offspring on *R*. *padi* after two and three generations of rearing on *R*. *padi*. This suggests there are recessive alleles for failure to oviposit or survive in *R*. *padi* that are slow to be removed by selection. *F*_1_ females from crosses between selection‐population males and control‐population females parasitized threefold more *R*. *padi* than control‐population females, but similar numbers to generation 3 selection‐population females. Backcross females from the cross of *F*_1_ females with control‐population males parasitized threefold more *R*. *padi* than control‐population females. *F*_1_ and backcross females parasitized more than two‐fold fewer *Aphis glycines* than control females, suggesting trade‐offs between parasitism of *R*. *padi* and *Aphis glycines*. Nonetheless, *F*_1_ females parasitized fivefold more *Aphis glycines* than *R*. *padi*, and backcross females parasitized three‐fold more *Aphis glycines* than *R*. *padi*. That the difference in parasitism of *R*. *padi* versus *Aphis glycines* is less for backcross females than for the *F*_1_ females is not surprising, given that about half of the backcross females should be homozygous for control alleles, whereas all *F*_1_ females should be heterozygous for control and selection alleles.

**TABLE 2:** Analysis of deviance in generalized linear models of parasitism of *Rhopalosiphum padi* by control, selection, *F*_1_ and backcross females of *Aphelinus rhamni*

Comparison	Model		Residual		*p*
	*df*	Deviance	*df*	Deviance	
Generation 2 of selection versus control	1	14.0	110	110.0	0.0002
Generation 3 of selection versus control	1	9.4	108	99.9	0.002
Generation 2 versus 3 of selection	1	1.7	184	188.4	0.20
*F*_1_, control and generation 3 of selection	2	6.0	111	81.3	0.05
*F*_1_: *Aphis glycines* versus *Rhopalosiphum padi*	1	64.6	55	83.5	<0.00001
Backcross versus control	1	5.7	410	278.9	0.02
Backcross: *Aphis glycines* versus *Rhopalosiphum padi*	1	36.4	492	424.4	<0.00001

The variance in parasitism of *R*. *padi* among selection‐population females declined from 9.4 aphids in generation 2 of selection to 5.1 aphids in generation 3 of selection (*F* = 1.8; *df* = 94,90; *p* = 0.004), presumably because of fixation of alleles involved in using the new host. The variance in parasitism among backcross females was 20.1 aphids and thus much larger than the variance of females from the control population (0.4 aphids) and the generation‐3 population (5.1 aphids) from which the backcross females were derived (Bartlett's K‐squared = 193.9, *df* = 2, *p* < 0.00001). Surprisingly, the variance in parasitism among *F*_1_ females (16.0) did not differ from the variance in parasitism among backcross females (*F* = 0.8; *df* = 56, 371; *p* = 0.29).

Assuming an initially linear response to selection, the response in parasitism of *R*. *padi* to selection over two generations selection was *R*_2_ = 1.9 aphids (i.e., the difference between the means of generation‐2 and control‐population means) and the selection differential over two generations was S1 + S2 = 1.95 aphids (i.e., 2 × 0.98, which is the difference between the selected‐parent mean and the unselected mean) so the narrow‐sense heritability can be estimated as 0.97, which is quite high and explains the rapid response to selection.

### 3.2 SNP locus–phenotype associations

We found with 9505 SNP loci in backcross females. Filtering these loci for those called in ≥150 backcross females left 3548 loci, filtering for nonduplicates left 2902 loci, and filtering for Mendelian segregation left 184 loci. Of these loci, 180 mapped to a single linkage group, which suggests that alleles of SNP loci that were fixed in selection versus control populations were concentrated on a single chromosome in *A*. *rhamni*.

**FIGURE 2:**
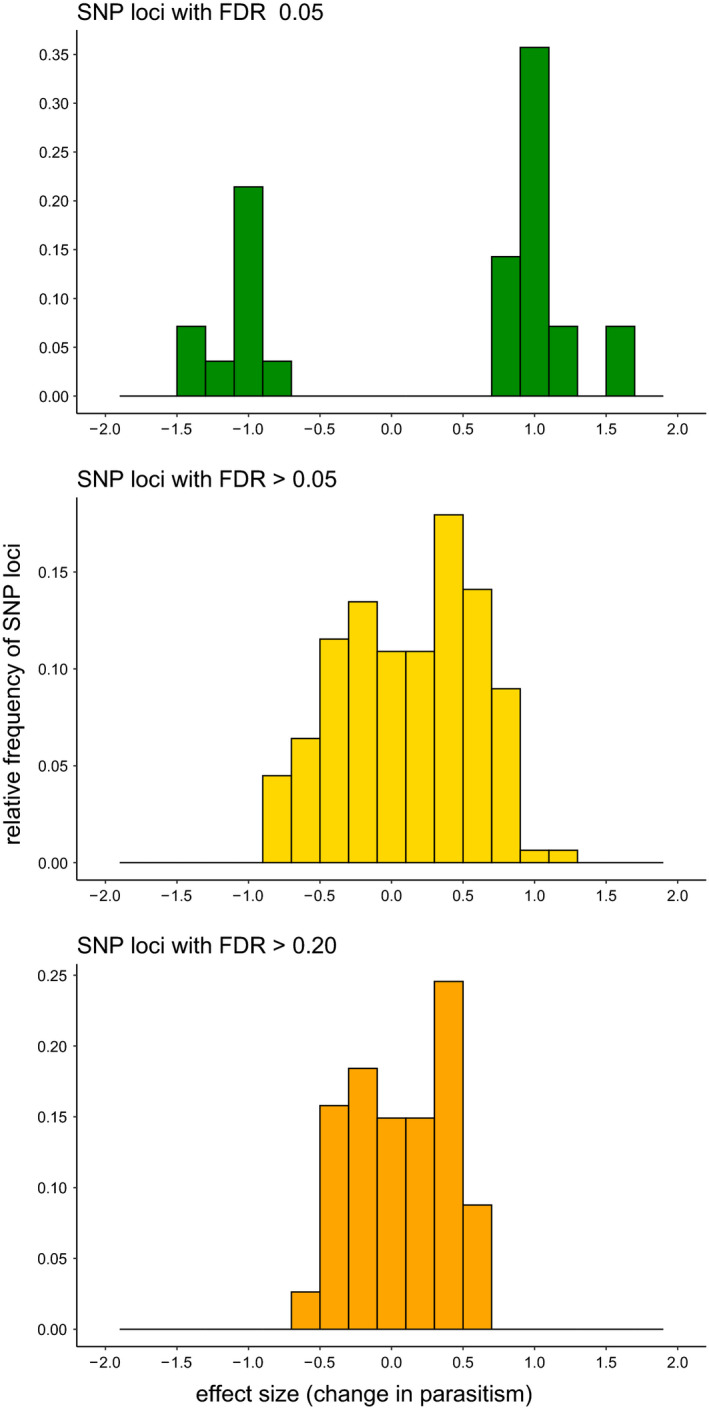
Effect sizes (difference in parasitism of *R*. *padi*) for SNP loci in backcross females of *Aphelinus rhamni* with FDR ≤0.05, FDR >0.05 and FDR >0.20

Among these 180 SNP loci, 28 (16 per cent) were associated with differences in parasitism of *R*. *padi* with FDR ≤0.05 (Table 3). Alleles in the selected population at 18 loci increased parasitism over control alleles by 0.8–1.6 aphids, and alleles at 10 loci decreased parasitism of *R*. *padi* by 0.9–1.3 aphids (Figure 2). The changes in parasitism per locus were similar in magnitude to the mean response to selection (Figure 2) and to the difference in mean parasitism between *F*_1_ and control females (Figure 1). Furthermore, together the effects of loci associated with parasitism could explain the large values seen in some backcross females. The 156 SNP loci that were not associated with parasitism of *R*. *padi*, that is with FDR >0.05, had small effect sizes, and the 114 SNP loci with FDR >0.20 had even smaller effect sizes (Figure 2). There was some overlap in effect sizes and raw probabilities between loci with FDR ≤0.05 and those with FDR >0.05, and we chose to compare candidate genes and functions of genes on contigs that had loci with FDR ≤0.05 with those that on contigs that had loci with FDR >0.20.

**TABLE 3:** Association between SNP loci and parasitism of *Rhopalosiphum padi* by *Aphelinus rhamni*

Contig in reference genome	SNP location on contig	Number scored	Mean difference in parasitism	Standard error difference in parasitism	*t*‐value	*p*	FDR
jcf7180003511679	10677	161	1.6	0.3	4.7	0.00001	0.0005
jcf7180003527363	68677	201	1.5	0.4	4.0	0.00009	0.003
jcf7180003484168	11876	256	1.2	0.3	3.9	0.0001	0.003
jcf7180003492397	33835	200	1.1	0.3	3.6	0.0005	0.01
jcf7180003566777	48842	165	1.1	0.3	3.4	0.001	0.01
jcf7180003500534	9974	198	1.0	0.3	3.3	0.001	0.01
jcf7180003515986	18427	231	1.0	0.3	3.0	0.003	0.03
jcf7180003519098	30786	156	1.0	0.3	2.8	0.01	0.04
jcf7180003533112	5283	308	1.0	0.3	3.5	0.001	0.01
jcf7180003481476	1738	282	1.0	0.3	3.3	0.001	0.01
jcf7180003567556	54017	251	1.0	0.3	3.3	0.001	0.01
jcf7180003528118	62740	255	0.9	0.3	3.3	0.001	0.01
jcf7180003541858	5886	174	0.9	0.3	2.9	0.004	0.03
jcf7180003547778	1048	302	0.9	0.3	3.7	0.0003	0.01
jcf7180003539937	169175	284	0.8	0.3	2.9	0.004	0.03
jcf7180003483396	36215	266	0.8	0.3	2.7	0.01	0.04
jcf7180003527384	21760	293	0.8	0.3	2.7	0.01	0.05
jcf7180003562350	35110	299	0.8	0.3	2.7	0.01	0.04
jcf7180003564987	4010	229	−0.9	0.3	−2.9	0.005	0.04
jcf7180003516028	9541	227	−0.9	0.3	−2.9	0.004	0.03
jcf7180003568593	2731	265	−0.9	0.3	−3.1	0.002	0.02
jcf7180003569262	3111	308	−0.9	0.3	−3.1	0.002	0.02
jcf7180003483293	2681	308	−0.9	0.3	−3.6	0.0003	0.01
jcf7180003484851	2241	212	−1.1	0.3	−4.1	0.0001	0.003
jcf7180003516500	9053	239	−1.1	0.3	−3.3	0.001	0.01
jcf7180003482154	714	237	−1.3	0.3	−4.1	0.00005	0.003
jcf7180003511540	2589	269	−1.3	0.3	−4.8	0.000002	0.0004
jcf7180003481639	5687	150	−1.3	0.3	−4.0	0.0001	0.003

### 3.3 Genome assembly and annotation

With 25 Gb of paired‐end Illumina reads that gave 52× coverage, our assembly of the *A*. *rhamni* genome was 384 Mb long and thus 20% smaller than the 483 Mb genome size estimated from flow cytometry (Gokhman et al., 2017). The difference in estimates of genome size between flow cytometry and assembly may result from repetitive DNA, which is difficult to assemble. Our assembly had an *N*_50_ of 18 Kb and 42k contigs with lengths ≥1 Kb. Despite the fragmentation of the assembly, it captured an almost complete set of insect genes, as measured by comparison with the 1658 genes in the BUSCO core insect‐gene set (Simão et al., 2015). Our assembly included 98 per cent of the core set, with 96 per cent complete genes, of which 95 per cent were single copy and 1 per cent were duplicated, 2 per cent of the core genes were fragmented, and only 2 per cent of the core genes were missing.

Using AUGUSTUS, we found 41,066 genes, whose combined length comprises 85 Mb of the genomic DNA sequence or 22 per cent of the assembly. Gene‐length mean is 2078 nucleotides, a mean of 3 exons per gene, exon‐length mean of 447 nucleotide and intron‐length mean of 611 nucleotides. Magic‐BLAST with RNAseq reads showed that 80 per cent of the genes were transcribed in adult females or males. BLASTP revealed homologs for 31,543 proteins (77 per cent of all proteins) among insects in the RefSeq database. The BLASTP homologs included 399 candidates that might affect host specificity, which comprise 124 venom proteins, 104 cytochrome p450 proteins, and 171 chemosensory proteins, including 60 odorant receptors, 21 gustatory receptors, 70 ionotropic receptors, and 20 odorant‐binding proteins. BLAST2GO showed gene‐ontology mappings for 28,645 genes (70 per cent of all genes) and gene‐ontology functional annotations for 20,954 genes (51 per cent of all genes). InterProScan identified functional domains in 32,908 genes (80 per cent of all genes).

### 3.4 Functions of genes near SNP loci associated with *R*. *padi* parasitism

**FIGURE 3:**
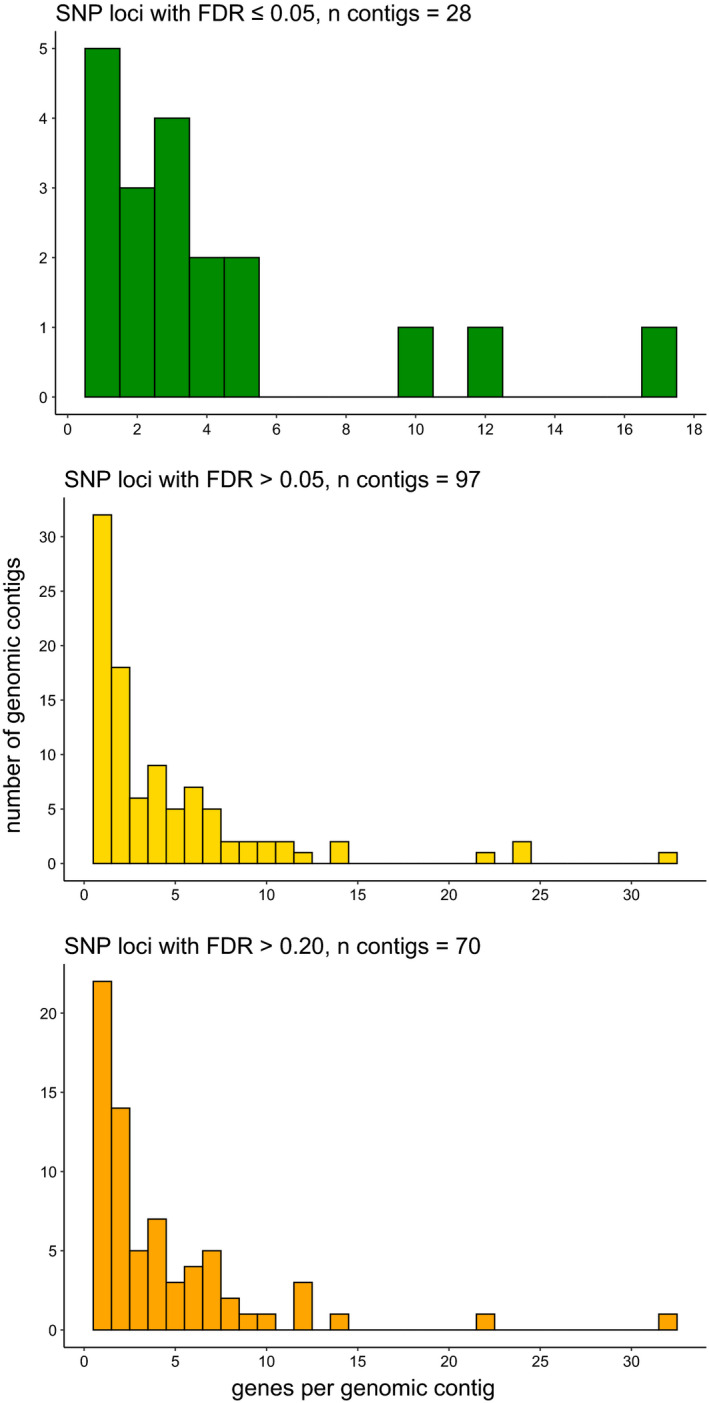
Numbers of genes per contig on contigs with SNP loci having FDR ≤0.05, FDR >0.05 and FDR >0.20 for association with parasitism of *Rhopalosiphum padi* by *Aphelinus rhamni* females from a backcross between selection populations reared for three generations on *R*. *padi* and control populations reared on *Aphis glycines*

We mapped the 184 SNP loci segregating between control and selection populations to 171 contigs in our draft *A*. *rhamni* genome assembly. We separated the contigs into three sets based on the false discovery rates of the SNP loci for association with parasitism of *R*.* padi*: FDR ≤0.05, FDR >0.05 or FDR >0.20, and these sets differed in effect sizes (Figure 2). Note that the FDR >0.05 set includes the FDR >0.20 set. The 28 SNP loci associated with parasitism of *R*. *padi* mapped to 28 contigs (Table 4), only one of which harboured more than one SNP locus. Together, these contigs comprised a total of 1.2 Mb, and as pointed out above, the loci and thus the contigs mapped to a single linkage group. The three sets of contigs had different numbers and lengths and harboured different numbers of genes (Table 4, Figure 3). We will not consider further the 56 contigs without genes. One long contig (167 kb) had two SNP loci, one of which was associated with parasitism of *R*. *padi* (FDR ≤0.05) and the other of which was not (FDR >0.05); however, the latter had *p* = 0.05 and effect size of 0.7, so we included the 10 genes from this contig in the category of those associated with parasitism of *R*. *padi*. Of the 28 contigs having loci associated with parasitism, 9 (median length = 16 kb) had no identified genes, but 19 (median length = 38 kb) harboured a total of 80 genes with most contigs having a single gene, a median of three genes per contig, and a maximum 17 genes per contig (Figure 3). Among these 80 genes, 74 had homologs among insect genes in the nr GenBank database, with all homologs in species of parasitic Hymenoptera, primarily *Nasonia vitripennis*. Furthermore, 69 genes could be assigned functional annotations with either BLAST2GO or InterProScan.

**TABLE 4:** Statistics from mapping SNP loci to *Aphelinus rhamni* genome assembly

FDR for effects of SNP loci on parasitism	*n* SNP loci	*n* genome contigs	*n* contigs with genes	Median length (kb)	*n* genes	*n* genes with blast hits	*n* genes with annotations
≤ 0.05	28	28	19	38	80	74	69
> 0.05	156	144	97	37	373	350	336
> 0.20	114	107	70	36	274	257	248
all loci	184	171	115	37	453	424	406

None of the 74 genes with insect homologs located on contigs with SNP loci associated with parasitism of *R*. *padi* were among the candidates expected to affect host specificity, that is those coding for chemosensory, venom or cytochrome p450 proteins. However, among the 373 genes on contigs with SNP loci that had FDR >0.05 for effects on parasitism, there were one odorant‐binding protein and three ionotropic receptors, one of which had an effect size of 0.9, *p* = 0.02 and FDR = 0.09 and so is a marginal candidate.

**FIGURE 4:**
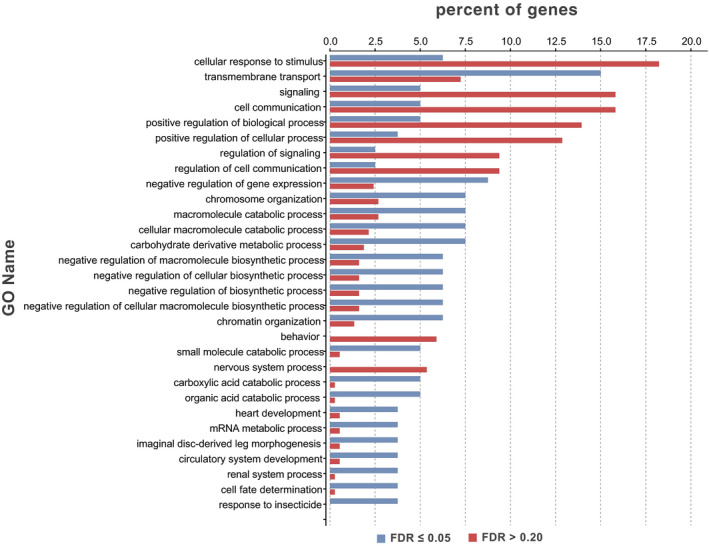
Biological processes of genes on contigs with SNP loci having FDR ≤0.05 versus FDR >0.20 for association with parasitism of *Rhopalosiphum padi* by *Aphelinus rhamni* females from a backcross between selection populations reared for three generations on *R*. *padi* and control populations reared on *Aphis glycines* (Fisher's exact test, *p* ≤ 0.05)

**FIGURE 5:**
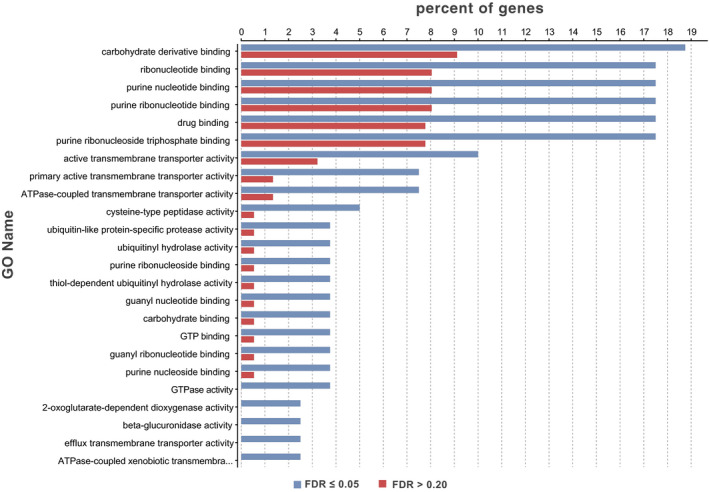
Molecular functions of genes on contigs with SNP loci having FDR ≤0.05 versus FDR >0.20 for association with parasitism of *Rhopalosiphum padi* by *Aphelinus rhamni* females from a backcross between selection populations reared for three generations on *R*. *padi* and control populations reared on *Aphis glycines* (Fisher's exact test, *p* ≤ 0.05)

We compared the biological processes and molecular functions of the set of genes on contigs with SNP loci‐associated differences in parasitism of *R*. *padi* (FDR ≤0.05) with those of the genes found on contigs with SNP loci definitely not associated with parasitism (FDR >0.20). Among biological processes, there were 18 specific processes and 41 more general processes that either increased or decreased among genes on contigs with SNP loci associated with parasitism compared with those that were not (Fisher's exact test: *p* ≤ 0.05; Figure 4). Among molecular functions, there were six specific functions and 11 more general functions that either increased or decreased among genes on contigs with SNP loci associated with parasitism (Fisher's exact test: *p* ≤ 0.05; Figure 5). However, with FDR = 0.05 to correct for multiple testing, we found no enrichment in biological processes or molecular functions between genes on contigs with SNP loci associated with parasitism of *R*. *padi* versus genes on contigs with SNP loci not associated with parasitism.

## 4 DISCUSSION

*Aphelinus rhamni* responded rapidly to selection for parasitism of *R*. *padi*, a suboptimal, low‐preference host. Selection for *R*. *padi* did not lead to parasitism at the level found with *Aphis glycines*, the original host of *A*. *rhamni*, but did lead to lower parasitism of this host by *F*_1_ and backcross females. There was no increase in parasitism of *R*. *padi* from generation 2 to generation 3 of selection, suggesting reduced variance available for selection. Indeed, even by generation 3 of selection, about 50 per cent of females failed to produce any progeny on *R*. *padi*, which suggests persistent, recessive alleles reducing parasitism of this aphid. The presence of dominance is also supported by expected versus observed means of *F*_1_ and backcross females. The expected value for parasitism of *R*. *padi* by *F*_1_ females is the average of the means for control and selection females, that is 1.06 aphids parasitized, but the observed value was 1.28 aphids parasitized, which is 21 per cent higher than the expected value. Furthermore, the expected value for parasitism of *R*. *padi* by backcross females is the average of the mean of control females and 50 per cent of the mean of selection females, that is 1.28 aphids parasitized, but the observed value was 1.47 aphids parasitized, which is 14 per cent higher than the expected value.

The increase in parasitism from rearing on *R*. *padi* is genetic rather than the result of conditioning, because *F*_1_ females parasitized five‐fold more *R*. *padi* than control females even though both sets of females were reared on *Aphis glycines*.

The genetic architecture of the response to selection appears complex, involving many genes that produce similar phenotypes, based on the effect sizes of the 28 SNP loci spread among 28 genomic contigs. However, all of these contigs mapped to a single linkage group and together comprised only 1.9 Mb, suggesting that a relatively small region of the *A*. *rhamni* 483 Mb genome was involved in the response to selection.

We identified 80 genes on the 19 contigs with SNP loci associated with *R*. *padi* parasitism, with the majority of these contigs having three or fewer genes. With one exception of an ionotropic receptor on a contig with a marginally significant SNP locus, none of these genes had annotations like those we expected, that is those coding for chemosensory, venom, or cytochrome p450 proteins. Furthermore, although there were differences in biological processes and molecular functions of these genes and those on contigs with SNP loci not associated with parasitism of *R*. *padi*, when a correction of multiple comparison was used, the differences disappeared.

However, there are several caveats concerning our results. First, although we found 28 SNP loci associated with *R*. *padi* parasitism on 28 contigs, our mapping population was on the small side, which limits the sampling of recombination events and the power to detect loci with small effects. All 184 SNP loci mapped to a single linkage group comprising 6.5 Mb. Some hitchhiking of regions with no effect on parasitism is likely, given that there had not been many meioses by generation 3. However, although all the SNP loci were in one linkage group and so on a single chromosome, some were associated with parasitism of *R*. *padi* and some were not, so there appears to have been sufficient recombination within the linkage group for loci to have segregated. Inability to detect small effects means we may not have identified all the loci associated with differences in parasitism. Another caveat concerns gene function. We found only 151 chemoreceptor proteins in *A*. *rhamni*. By contrast, *Nasonia vitripennis*, a parasitoid with a well‐annotated genome that is smaller than that of *A*. *rhamni* based on flow cytometry estimates (Gokhman et al., 2017), has a rich complex of 272 chemoreceptor proteins (Robertson et al., 2010). There may be more chemoreceptor genes in *A*. *rhamni* species, but because such genes evolve rapidly, they can be difficult to identify by homology‐based searches (Sanchez‐Gracia et al., 2009). Thus, additional chemoreceptor genes may exist among those without blast hits or gene ontology annotations. Another and perhaps more valid approach to determining function would be to analyse tissue‐ or cell‐specific expression of candidate genes of unknown function, which we are pursuing.

These results provide one of the few studies of the genetic architecture of host specificity in parasitic wasps. However, research on host specificity in *Nasonia* species indicates a similar architecture, where a single region of the genome explained differences in host specificity between *Nasonia vitripennis*, a generalist, and *Nasonia giraulti*, a specialist. Desjardins et al. (2010) identified a 16‐Mb region that when introgressed from *N*. *vitripennis* into *N*. *giraulti*, altered its host specificity, and more precise mapping has further delineated this to a 4.1‐Mb region (Leung, 2020).

## 5 CONCLUSIONS

The implications of our results for the evolutionary shifts of host specificity are somewhat equivocal. Although there was a rapid response to selection for parasitism of *R*. *padi*, the levels remained much lower than those for parasitism of its original host, *Aphis glycines*, with *F*_1_ females of the cross between selection and control populations parasitizing five‐fold fewer *R*. *padi* than *Aphis glycines*. We have continued to rear the selection populations on *R*.* padi*, and preliminary results after over 140 generations of selection show that the selection population females still parasitize less than half as many *R*. *padi* as *Aphis glycines*.

## CONFLICT OF INTEREST

The authors declare no conflict of interest related to this manuscript.

## Data Availability

Data from the host adaptation experiments are archived on Ag Data Commons (DOI: https://doi.org/10.15482/USDA.ADC/1522563). The DNA sequence data are archived at NCBI (BioProject PRJNA733215).
